# *EML4-ALK*融合基因阳性肺多原发神经内分泌癌1例

**DOI:** 10.3779/j.issn.1009-3419.2025.102.10

**Published:** 2025-03-20

**Authors:** Yin ZHANG, Yue HOU, Tianming ZHANG, Hong WANG

**Affiliations:** 730030 兰州，兰州大学第二医院呼吸与危重症医学科; Department of Pulmonary and Critical Care Medicine, Lanzhou University Second Hospital, Lanzhou 730030, China

**Keywords:** 肺肿瘤, 肺大细胞神经内分泌癌, ALK突变, 靶向治疗, Lung neoplasms, Large cell neuroendocrine carcinoma of the lung, ALK mutation, Targeted therapy

## Abstract

神经内分泌癌（neuroendocrine carcinoma, NEC）属于高增殖活性的神经内分泌肿瘤，具有侵袭性强、预后差等特点。本文报道1例既往健康无吸烟史的女性患者，其先后在不同侧肺叶发生NEC，苏木精-伊红（hematoxylin-eosin, HE）染色病理诊断分别为肺大细胞神经内分泌癌（large cell neuroendocrine carcinoma, LCNEC）与小细胞肺癌（small cell lung cancer, SCLC），分别对上述病变组织进行二代测序（next-generation sequencing, NGS），结果均存在棘皮动物微管相关蛋白4-间变性淋巴瘤激酶（echinoderm microtubule-associated protein-like 4-anaplastic lymphoma kinase, EML4-ALK）融合突变，且患者在ALK-酪氨酸激酶抑制剂（tyrosine kinase inhibitors, TKIs）靶向治疗中取得了显著的治疗效果。

## Abstract (en)

Neuroendocrine carcinoma (NEC), a subtype of neuroendocrine tumors with high proliferative activity, is characterized by strong invasiveness and poor prognosis. This article reports a previously healthy female non-smoker who developed NEC occurring sequentially in different lobes of both lungs. The lesions were pathologically diagnosed by hematoxylin-eosin (HE) staining as large cell neuroendocrine carcinoma (LCNEC) and small cell lung cancer (SCLC), respectively. Next-generation sequencing (NGS) performed on both lesions revealed the presence of echinoderm microtubule-associated protein-like 4-anaplastic lymphoma kinase (EML4-ALK) fusion mutations in both lesions. Notably, the patient achieved a significant therapeutic response to ALK-tyrosine kinase inhibitors (TKIs) targeted therapy.

2021年世界卫生组织将典型类癌（typical carcinoid, TC）、非典型类癌（atypical carcinoid, AC）与小细胞肺癌（small cell lung cancer, SCLC）以及肺大细胞神经内分泌癌（large cell neuroendocrine carcinoma, LCNEC）均归为神经内分泌癌（neuroendocrine carcinoma, NEC）^[1]^。其中，LCNEC和SCLC均为恶性程度高、易转移且预后较差的肿瘤。患病群体多为有长期吸烟史的老年男性^[2]^。因肿瘤易复发和转移，并且易出现对化疗或靶向药物耐药，所以预后较差。LCNEC目前仍无标准治疗指南，主要借鉴SCLC的治疗方式。SCLC的主要治疗方式包括手术和化疗，但化疗对LCNEC患者疗效有限，亟待新的治疗方案来提高患者生活质量、延长患者生存期。

近年来随着二代测序（next-generation sequencing, NGS）的发展与创新，我们得以系统分析肿瘤的分子特征，发现可用于靶向治疗的驱动基因突变以及更精准的生物标志物，为肿瘤的精准治疗、药物研发以及耐药性机制研究奠定了基础。George等^[3]^根据LCNEC分子水平差异将其分为：具有TP53、STK11/TEAK1突变的I型和具有TP53和RB1双失活的II型。将肿瘤进行分子水平细分类有利于准确区分I型、II型LCNEC与SCLC，揭示可能的靶向治疗靶点。因此临床可对肿瘤做出更加系统、全面的基因组肺癌诊断，从而实现肿瘤的分层靶向治疗。

本文报道1例先后确诊右肺中下叶LCNEC及左主支气管SCLC的病例资料，通过对该病例的总结分析，期待能进一步提高临床医生对多原发肺癌（multiple primary lung cancer, MPLC）的认知，为临床评估LCNEC和SCLC患者靶向治疗潜力提供新参考。

## 1 病例资料

患者，女，61岁，既往无吸烟饮酒史及特殊物质接触史，2019年3月26日因肺部占位（图1）于外院行“右肺中下叶切除+系统淋巴结清扫+胸腔闭式引流术”。术中见肿瘤位于右肺下叶支气管开口处，大小约4 cm×3 cm。术后病理提示为低分化LCNEC伴3、7、11组淋巴结转移，肿瘤术后分期为：T2aN2M0，IIIA期。免疫组织化学染色（immunohistochemistry, IHC）：甲状腺转录因子1（thyroid transcription factor 1, TTF-1）（+）、新天冬氨酸蛋白酶（new aspartic proteinase A, Napsin A）（-）、Vimentin（-）、广谱细胞角蛋白（cytokeratin-pan, CKp）（+）、CK5/6（-）、CK7（+）、P40（-）、突触核蛋白（synuclein, Syn）（＋）、嗜铬粒蛋白A（chromogranin A, CgA）（+）、CD56（＋）、Ki-67（增殖指数：60%）、间变性淋巴瘤激酶（anaplastic lymphoma kinase, ALK）（+）（图2）。NGS提示存在棘皮动物微管相关蛋白样4（echinoderm microtubule-associated protein-like 4, EML4）-ALK融合突变。患者术后依据指南推荐接受“紫杉醇+洛铂”化疗联合克唑替尼250 mg bid。化疗1个周期后，患者出现严重消化道不良反应，遂停止化疗，继续口服克唑替尼靶向治疗。患者规律口服克唑替尼36个月，至2022年4月因出现严重药物性皮疹而停用克唑替尼。服药期间患者规律复查，病灶稳定，未见肿瘤术后复发及靶向药物所致间质性肺炎（图1）。 2023年8月患者再次于外院复查正电子发射计算机断层扫描（positron emission tomography/computed tomography, PET/CT）示：左主支气管略狭窄（图3）。2023年10月8日就诊于我院行胸部增强CT示：右肺术后改变，气管隆突及左主支气管壁明显增厚、管腔略窄（图3）。支气管镜检查示：右中下叶术后残端光滑，左主支气管新生物阻塞管腔约50%（图4）。支气管镜下取活检，病理提示为SCLC，肿瘤分期为局限期。IHC：TTF-1（+）、Napsin A（-）、CK7（+）、CKp（-）、CK5/6（-）、P63（-）、P40（-）、Syn（＋）、CgA（-）、CD56（＋）、LCA（-）、Ki-67（增殖指数: 70%）（图5）。NGS提示存在EML4-ALK融合突变，突变频率为12.25%；RB1点突变，突变频率为51.17%；肿瘤突变负荷（tumor mutational burden, TMB）：1.43 Muts/Mb；微卫星不稳定性（microsatellite instability, MSI）：MSI-L/MSS。患者自2023年10月14日开始，依据指南推荐行“依托泊苷+顺铂”方案化疗联合阿来替尼600 mg bid。化疗3个周期后，患者出现严重消化道不良反应，遂停止化疗，继续口服阿来替尼靶向治疗。2024年5月即服用阿来替尼7个月后，复查胸部CT平扫示：左主支气管基本通畅（图3），腹部CT平扫、全身骨显象、头颅核磁均未见异常。支气管镜检查示：右中下叶手术残端黏膜光滑，左主支气管管腔通畅，黏膜较前明显好转，未见阻塞、出血及新生物。疗效评估为完全缓解（complete response, CR）（图4）。2024年10月，即服用阿来替尼12个月后复查胸腹部CT（图3）、单光子发射计算机断层扫描（single photon emission computed tomography, SPECT）全身骨显象均未发现疾病新进展。患者病情持续稳定，目前仍定期我科随访中。

**图1:**
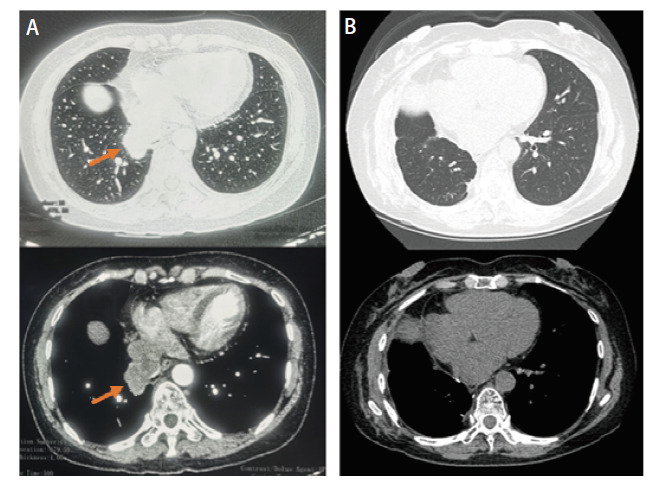
大细胞神经内分泌癌影像学表现。A：2019-03-19术前胸部CT：肺窗（上）、纵隔窗（下），可见右肺下叶内基底段及右肺门软组织占位；B：2022-04-27胸部CT：肺窗（上）、纵隔窗（下），未见肿瘤复发及转移，未见间质性肺炎等药物不良反应。

**图2:**
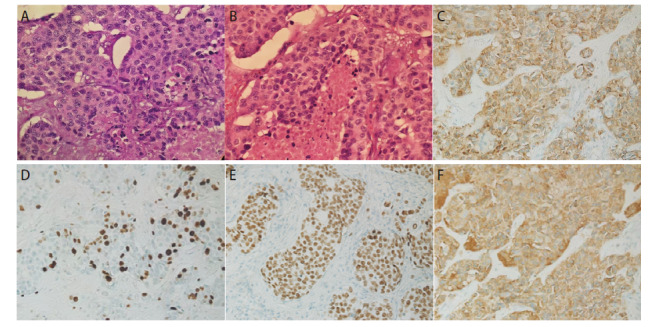
肺组织病理活检结果（2019-03-27）。A：大的多角形癌细胞呈片状或巢状分布，密集排列。细胞核大，染色质呈空泡状，可见小核仁，胞质丰富、红染（HE染色，×200）；B：癌细胞巢中央可见大量凝固性坏死（HE染色，×200）；C：肿瘤组织TTF-1（+）（SP法，×200）；D：肿瘤组织Syn（+）（SP法，×200）；E：肿瘤组织CgA（+）（SP法，×200）；F：肿瘤组织Ki-67增殖指数60%（SP法，×200）。

**图3:**
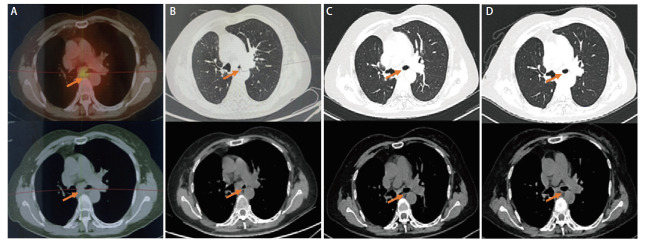
小细胞肺癌影像学表现。A：2023-08-28 PET/CT：左主支气管略狭窄；B：2023-10-08胸部CT：肺窗（上）、纵隔窗（下）可见左主支气管较2023-08-28 PET/CT狭窄程度明显加重；C：2024-05-13即服用阿来替尼7个月后复查胸CT：肺窗（上）、纵隔窗（下），可见左主支气管狭窄明显改善；D：2024-10-23即服用阿来替尼12个月后复查胸部CT：肺窗（上）、纵隔窗（下），未见疾病进展。

**图4:**
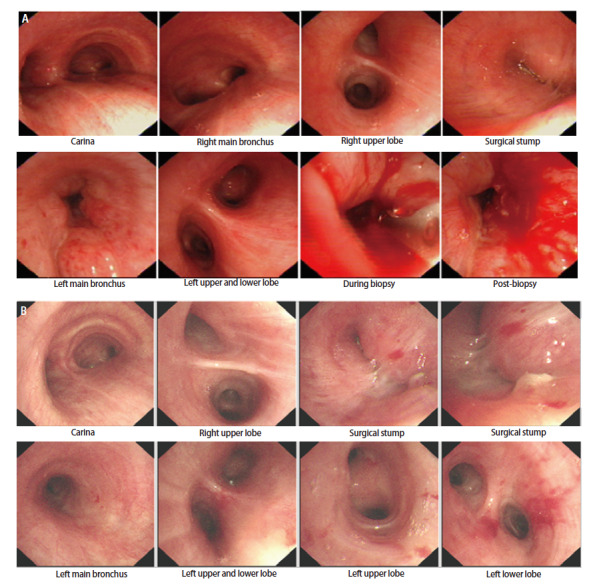
支气管镜检查结果。A：2023-10-10支气管镜检查示：右中下叶手术残端黏膜光滑，左主支气管新生物阻塞管腔约50%，新生物沿管壁呈浸润性生长、肿物表面凹凸不平，活检质脆易出血，左上下叶未见阻塞、出血及新生物；B：2024-05-14即服用阿来替尼7个月复查支气管镜示：右中下叶手术残端黏膜光滑，左主支气管管腔通畅，左上下叶支气管管腔及黏膜正常。

**图5:**
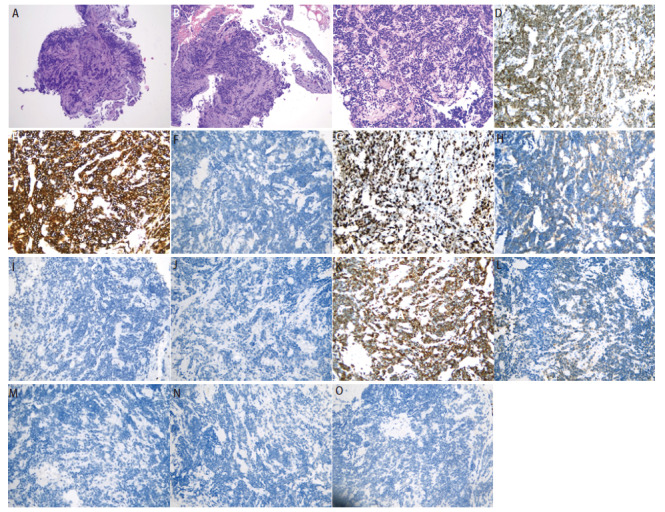
支气管组织病理活检结果（2023-10-13）。A：支气管活检组织（HE染色，×100）；B：支气管活检组织（HE染色，×100）；C：支气管活检组织（HE染色，×200）：HE染色可见肿瘤细胞呈巢状、梁索状及团簇状排列，细胞呈圆形或卵圆形，部分核形不规则，胞浆稀少，染色质呈细颗粒状，核分裂可见。D：肿瘤组织 TTF-1（+）（SP法，×200）；E: 肿瘤组织Syn（+）（SP法，×200）；F：肿瘤组织CgA（-）（SP法，×200）；G：支气管活检组织，肿瘤细胞Ki-67增殖指数70%（SP法，×200）；H：肿瘤组织CD56（+）（SP法，×200）；I：肿瘤组织LCA（-）（SP法，×200）；J：肿瘤组织CK5/6（-）（SP法，×200）；K：肿瘤组织CK7（+）（SP法，×200）；L：肿瘤组织CKp（-）（SP法，×200）；M：肿瘤组织Napsin A（-）（SP法，×200）；N：肿瘤组织P40（-）（SP法，×200）；O：肿瘤组织P63（-）（SP法，×200）。

回顾患者病程， 第一右肺中下叶原发肿瘤自2019年4月随访至2023年8月，患者无进展生存期（progression-free survival, PFS）为53个月，第二支气管原发肿瘤自2023年10月随访至2025年1月，患者PFS为16个月。总生存期（overall survival, OS）超过69个月。

## 2 讨论

本文报道1例肺癌根治术后再次出现第二原发支气管肿瘤的病例，回顾本病例的诊治过程，有以下几点值得我们关注。

首先，本文患者两次肿瘤病灶所处解剖位置不同、肿瘤组织学类型不同且间隔时间>4年，4年内复查均未见手术残端肿瘤复发及转移，根据Martini^[4]^和美国胸科医师协会（American College of Chest Physicians, ACCP）2013年的诊断标准^[5]^，可诊断为异时性多原发肺癌（metachronous MPLC, mMPLC）。MPLC是指同时或先后在肺内发现2个或者2个以上的原发肺癌病灶，根据两次肿瘤出现的时间间隔可将其分为同时性MPLC（synchronous MPLC, sMPLC）和mMPLC。近年来随着影像学、组织病理学、分子生物技术、人工智能识别诊断技术的不断发展和广泛应用，MPLC的检出率逐年增高。临床中，不同的组织学类型是诊断MPLC较为可靠的依据，但当肿瘤组织学类型相同时，MPLC的诊断往往需要与原发肺癌肺内转移（intrapulmonary metastasis, IPM）进行鉴别，准确的诊断对确定肿瘤分期和选择治疗方案至关重要^[6]^。

MPLC可表现出独特的分子突变机制，故可通过检测表皮生长因子受体（epidermal growth factor receptor, EGFR）、ALK、p53的突变状态来与IPM进行鉴别。2007年的一项研究^[7]^表明MPLC中存在更高频率的EGFR突变。除了检测单独的突变基因，列阵比较基因组杂交（comparative genomic hybridization, CGH）分析可以帮助判断多个同时性肿瘤之间的关系^[8]^。当病理标本较小、不足以全面了解肿瘤内部成分时，还可以通过检测多个肿瘤的突变谱，并根据其差异来判断。即使肿瘤每个部位的组织学可能不同，但主要的突变仍会存在于同一克隆的肿瘤中^[9]^。同时，对检测到的大量基因数据按照相似性分组即聚类分析，有助于评估多克隆癌症病灶中癌症的克隆进展。即使两个肿瘤之间的部分突变谱在某种程度上匹配，也可以通过进行聚类分析来估计它们之间的遗传距离，从而推测肿瘤是原发性或转移性^[10]^。

影像学资料也有助于鉴别诊断，出现以下影像学特点时更倾向于诊断为IPM：（1）实性为主的磨玻璃结节或纯实性结节；（2）CT上未显示毛刺或支气管充气征；（3）不同病灶之间大小差异较大。虽然分叶状结节在IPM与MPLC中均多见，但是MPLC的分叶更加明显。同时，IPM更容易出现淋巴结受累也可作为诊断参考^[11]^。

虽然MPLC的诊断标准在不断完善和更新，但以上方法都有一定的局限性，并不能普遍适用于所有患者，ACCP认为目前基于分子生物学的诊断标准均只能作为参考^[5]^，临床仍缺少普遍适用的统一的诊断标准。

其次，本文患者在LCNEC和SCLC中均发现了EML4-ALK融合突变。该融合基因多见于非小细胞肺癌（non-small cell lung cancer, NSCLC），特别是肺腺癌，是一种新型分子靶向治疗靶点。2007年Soda等^[12]^第一次报道了EML4-ALK融合基因，并在后续实验中发现，EML4-ALK转基因小鼠在出生仅几周便出现双肺腺癌结节且结节迅速生长，证实了EML4-ALK具有致癌活性。Soda等^[13]^随后给予转基因小鼠口服ALK-TKIs治疗，发现ALK-TKIs虽不能使肿瘤细胞完全消失，但可显著降低小鼠的肿瘤负荷并提高动物的存活率。随着第一代ALK-TKIs克唑替尼进入临床，已有诸多研究证明，ALK阳性患者接受ALK-TKIs新辅助治疗或术后辅助治疗，可有效降低肿瘤术前分期、改善预后、降低复发风险。有研究^[14]^表明，在未经治疗的ALK阳性晚期NSCLC患者中，与培美曲塞联合铂类药物的化疗相比，克唑替尼作为一线治疗时患者PFS显著延长。第二代ALK-TKIs，如：阿来替尼，相较于克唑替尼，其血脑屏障（blood-brain barrier, BBB）的透过率更高，在中枢神经系统（central nervous system, CNS）的活性更高。2022年报道了1例无吸烟史的ALK阳性LCNEC患者，其在脑转移灶切除术后服用阿来替尼靶向治疗，PFS超过10个月^[15]^。尽管第二代ALK-TKIs疗效优于第一代，但耐药、颅内转移等严重影响患者生存及预后的事件仍有发生。第三代ALK-TKIs如：劳拉替尼，则可覆盖更多的耐药突变，对第二代ALK-TKIs治疗后最常见的耐药突变ALK G1202R治疗有效，同时还具有更高的BBB穿透率，在脑转移的预防和治疗方面效果显著，可用于既往ALK-TKIs治疗失败的患者。但劳拉替尼治疗过程中出现3或4级不良事件的可能性也明显高于克唑替尼，患者更易出现高脂血症、水肿以及认知障碍等情况^[16]^。在临床中需结合患者病情及耐受情况合理选择药物。

与NSCLC相比，LCNEC与SCLC的驱动基因变异阳性率较低，ALK突变在LCNEC与SCLC中极为罕见。查阅以往报道病例可以发现ALK突变多见于无吸烟史的年轻女性患者且ALK-TKIs在ALK突变的NEC的治疗中展现出一定的潜力。2023年报道了1例无吸烟史的ALK阳性LCNEC女性患者，在服用阿来替尼治疗后获得了长达32个月的PFS，且治疗过程中并未报道严重药物不良反应^[17]^。鉴于ALK突变在LCNEC与SCLC中的低发生率，故临床医生可筛选出靶向治疗获益可能性高的患者，推荐其进行基因检测，化疗联合靶向治疗或将改善治疗效果，延长患者的生存期。
